# Adverse childhood experiences and burnout among health care providers in primary care: the moderating role of resilience

**DOI:** 10.1017/S1463423626100851

**Published:** 2026-03-02

**Authors:** Emma C. Lathan, Madeline Cohodes, Hailie R. Suarez-Rivas, Ryan A. Langhinrichsen-Rohling, Vedaja Surapaneni, Tamara Haynes, Stan C. Sonu, Abigail Powers

**Affiliations:** 1 Department of Psychological Sciences, Auburn University, Auburn, AL, USA; 2 Emory University School of Medicine, Atlanta, GA, USA; 3 Department of Psychiatry and Behavioral Sciences, Emory University School of Medicine, Atlanta, GA, USA; 4 Department of Psychiatry and Behavioral Sciences; General Medicine and Geriatrics, Emory University School of Medicine, Atlanta, GA, USA; 5 Department of General Medicine and Geriatrics, General Pediatrics and Adolescent Medicine, Emory University School of Medicine, Atlanta, GA, USA

**Keywords:** adverse childhood experiences, burnout, resilience

## Abstract

Health care providers (HCPs) with histories of adverse childhood experiences (ACEs) are at increased risk for burnout, which can threaten healthcare quality. This study examines the relation between ACEs and burnout among HCPs in primary care clinics at a safety-net hospital and whether this association is buffered by resilience. Sixty-seven HCPs (68.7% women; 44.8% White; *M*_*age*_ = 36.7 years, *SD*_*age*_ = 9.8) recruited from a large, public U.S. healthcare system participated in an anonymous study assessing their ACE history, resilience, and burnout symptoms. ACE scores were positively correlated with burnout, *r* =.25, *p* =.048. A moderation analysis revealed main effects of ACEs, *B* = .17, *SE* = .07, *p* = .013, and resilience, *B* = −.34, *SE* = .08, *p* = .000, on HCP burnout, when controlling for years in healthcare. ACEs and resilience interacted to predict burnout, *n* = 55, *B* = −.11, *SE*=.05, *p* = .029. A positive relation was found between ACEs and burnout for HCPs who reported low, *t* = 3.21, *p* = .002, and average, *t* = 2.57, *p =* .013, resilience levels. Resilience appears to mitigate, or even prevent, burnout among HCPs, although it may be most helpful for those with ACE histories. Healthcare systems can build a more resilient workforce by offering routine, system-wide exposure to trauma-informed professional development or self-care opportunities to their HCPs.

Burnout, or a psychological response to chronic work-related stress characterized by feelings of emotional exhaustion, depersonalization, and diminished personal accomplishment (Maslach *et al*., 1997), is a serious occupational hazard associated with far-reaching health and societal consequences (Bridgeman e*t al*., 2018; Han *et al*., 2019). Health care providers (HCPs) are at particular risk of burnout (West *et al*., 2018) given the emotional intensity and demanding nature of their work (Agency for Healthcare Research and Quality, 2023), the evolving landscape of care provision due to technological innovation (i.e., electronic health records, new delivery approaches, physician order entry) (Shanafelt *et al*., 2016; Dyrbye *et al*., 2017), and the increased likelihood of burnout associated with long hours and shift work (Wisetborisut *et al*., 2014; Robertson *et al*., 2016). Since the COVID-19 pandemic, HCP burnout rates have reached ‘crisis’ levels (Nigam *et al*., 2023), with one in every two providers experiencing burnout often (Yellowlees *et al*., 2021). HCPs at safety-net hospitals, broadly defined as publicly funded hospitals that provide care regardless of a patient’s insurance status or ability to pay (Popescu *et al*., 2019), may be at even greater burnout risk since they face unique challenges associated with caring for underserved communities: they see high acuity patients with multiple comorbidities and barriers to care (Figueroa and Jha, 2018). High levels of HCP burnout are concerning in that they threaten healthcare quality (Khullar, 2023) and safety (Dewa *et al*., 2014); specifically, burnout is associated with increased medical errors (Tawfik *et al*., 2018; Owoe *et al*., 2022; Li *et al*., 2023), reduced patient satisfaction (Anagnostopoulos *et al*., 2012), and poorer clinical outcomes (Mangory *et al*., 2021). Patients seeking care from safety-net hospitals have limited care options, therefore understanding factors that increase or mitigate the risk of burnout among HCPs in public healthcare systems is critical.

Research has consistently demonstrated a link between adverse childhood experiences (ACEs), defined as experiences of abuse, neglect, or household dysfunction before the age of 18 (Felitti *et al*., 1998), and burnout among physicians (Yellowlees *et al*., 2021) and other healthcare professionals (Clemens *et al*., 2021; Williams *et al*., 2021; Williamson *et al*., 2025). On average, half of HCPs report experiencing one or more ACEs (Stork *et al*., 2020; Mercer *et al*., 2023). Notably, HCPs with higher childhood adversity scores report greater levels of compassion fatigue (Renkiewwicz and Hubble, 2021) and miss more days of work (Maunder *et al*., 2010). In addition, a recent study found that emergency medical service (EMS) professionals with personal histories of ACEs were at increased risk of vicarious traumatization (i.e., trauma symptoms resulting from exposure to traumatic experiences of patients; Renkiewicz and Hubble, 2023), opportunities for which are abundant in the medical profession. Considering the negative impacts of ACEs and burnout on HCPs’ personal and professional well-being, identifying strategies to minimize their effects and promote patient-centred, trauma-informed care is of utmost importance.

One factor that may buffer against ACEs’ impact on HCP burnout is resilience. While ongoing debate exists regarding its definition (Southwick *et al*., 2014), there is consensus that resilience involves the ability to successfully adapt or ‘bounce back’ from adversity (Connor and Davidson, 2003). Resilience has been found to buffer against burnout in Portuguese nurses and doctors (Ferreira *et al*., 2021), Australian general practitioner trainees (Cooke *et al*., 2013), and medical staff within a National Health Service Trust in the United Kingdom (McCain *et al*., 2018). However, no studies have examined resilience’s potential protective role against burnout in the context of ACEs among HCPs. Thus, the current study (i) examines the association between ACEs and burnout in a sample of HCPs at a public healthcare system and (ii) explores whether this association is moderated by resilience. We hypothesize (H1) there will be a positive association between ACEs and burnout, and (H2) ACEs and resilience will interact to predict burnout, such that the association between ACEs and burnout will be attenuated by resilience.

## Method

### Participants and procedure

As part of a primary care-wide trauma-informed care initiative, HCPs at a public teaching hospital in a large city in the southeastern United States were recruited via electronic and paper flyers to complete an online survey assessing their trauma history and professional well-being. See Lathan *et al*. (2023) and Powers *et al*. (2024) for more information. For inclusion, participants must have provided direct patient care (i.e., physician/resident, nurse, medical assistant) within a primary care clinic at Grady Memorial Hospital at the time of the study, been able to read, speak, and understand English and been willing and able to participate in a research study and provide informed consent.

The authors assert that all procedures contributing to this work comply with the ethical standards of the relevant national and institutional guidelines on human experimentation (i.e., Emory University Institutional Review Board and the Grady Research Oversight Committee) and with the Helsinki Declaration of 1975, as revised in 2008. After following the survey link/QR code in the recruitment advertisement, the study’s purpose was presented via a REDCap page. Electronic consent was required before proceeding to the survey items. Participants could opt out of answering any item. At the end of the survey, participants were debriefed, compensated $25, and offered a list of community resources. Sixty-seven HCPs completed the survey between May 2022 and August 2022 (68.7% women; 44.8% White; *M*_*age*_ = 36.7 years, *SD*_*age*_ = 9.8; Table 1).

**Table 1.** Sample characteristics

Variable	% (*n*)
**Race**	
White	44.8 (*n* = 30)
Black/African American	29.9 (*n* = 20)
Asian/Asian American	17.9 (*n* = 12)
Multiracial	6.0 (*n* = 4)
Other	1.5 (*n* = 1)
**Ethnicity**	
Not hispanic or hatine	88.1 (*n* = 59)
Hispanic or latine	9.0 (*n* = 6)
Missing	3.0 (*n* = 2)
**Gender**	
Woman	68.7 (*n* = 46)
Man	28.4 (*n* = 19)
Non-binary	1.5 (*n* = 1)
Missing	1.5 (*n* = 1)
**Occupation**	
Physician or resident	74.6 (*n* = 50)
Medical assistant	17.9 (*n* = 12)
Nurse	7.5 (*n* = 5)
**Adverse childhood experiences (ACEs)**	
None	40.3 (*n* = 27)
One	28.4 (*n* = 19)
Two	10.4 (*n* = 7)
Three	10.4 (*n* = 7)
Four or more	7.5 (*n* = 5)
Missing	3.0 (*n* = 2)
**Burnout**	
Very low burnout	38.8 (*n* = 26)
Danger signs of burnout	40.3 (*n* = 27)
Clinically significant burnout	16.4 (*n* = 11)
Very serious problem of burnout	4.5 (*n* = 3)
Immediate professional help needed	0.0 (*n* = 0)
Variable	***M*** **(** ***SD*** **), Range**
**Age in years**	36.7 (9.8), 27.00–64.00
**Number of years in the healthcare profession**	7.0 (7.4), 1.00–32.00

### Measures

#### Demographics

HCPs were asked to provide various demographic characteristics, including race, ethnicity, gender, occupation, and age. We also assessed healthcare-related experience via the following item: ‘How many years have you been working in the healthcare profession?’

#### ACEs

ACEs were assessed using the ACE Questionnaire (Felitti *et al*., 1998), a 10-item self-report measure assessing exposure to various forms of abuse, neglect, and household dysfunction before the age of 18. Each item is rated on a binary scale (0 = *No*, 1 = *Yes*) and items are summed to generate a total score, with higher scores indicating greater ACE exposure.

#### Resilience

The Connor-Davidson Resilience Scale-2 (CD-RISC-2; Vaishnavi *et al*., 2007), a 2-item self-report instrument developed as a brief version of the CD-RISC, was used to measure providers’ resilience (i.e., their ability to adapt and bounce back after stress). Responses are rated on a 0 (*Not true at all*) to 4 (*True nearly all the time*). Items were summed to generate a total score with a range of 0 to 8. Greater scores suggest higher levels of resilience. Cronbach’s alpha indicated good internal consistency (α = .86).

#### Burnout

Burnout was measured via the Burnout Measure, Short Version (BMS; Malach-Pines, 2005), a unidimensional 10-item assessment of physical, emotional, and mental exhaustion. Participants were asked, ‘When you think about your work overall, how often do you feel the following?’ in relation to a range of burnout symptoms (e.g., trapped, ‘I’ve had it’). Responses are rated on a 1 (*Never*) to 7 (*Always*) scale. The BMS, an abbreviated version of the 21-item Burnout Measure (Pines and Aronson, 1988), has demonstrated validity and reliability among nurses (Malach-Pines, 2005) and physicians (Alrawashdeh *et al*., 2021). Mean scores are reported. Mean scores can indicate very low burnout (<2.4), danger signs of burnout (2.5–3.4), burnout (3.5–4.4), very serious problem of burnout (4.5–5.4), and immediate professional help needed (>5.5). Internal consistency was excellent (α = .90).

### Data analyses

There were no missing burnout data. Two participants (3.0%) failed to respond to any ACEs items; these participants were removed from further analyses. Three participants (4.4%) had one ACE item missing; available data were summed to create a total score. Roughly 11.9% (*n* = 8) of resilience scores were missing. Skewness (.45–1.73) and kurtosis (.31–3.47) fell within normal ranges.

Descriptive statistics were conducted to identify rates of ACEs and burnout among the HCPs. A one-way analysis of variance was conducted with post-hoc LSD tests to examine mean differences in ACEs, burnout, resilience, and years of experience by occupation (physician/resident vs. medical assistant vs. nurse). A correlation matrix was generated to examine the bivariate relations among HCPs’ ACEs, resilience, burnout, and years of experience scores. Pairwise deletion was used, so sample sizes differ across analyses. Moderation models were then conducted using Hayes’ PROCESS Macro to assess for interaction effects of ACEs and resilience on burnout. ACEs was entered as the independent variable, burnout as the dependent variable, resilience as the moderator, and number of years in the healthcare field was entered as a covariate, given its potential association with burnout (Meredith *et al*., 2022). All variables were mean-centred. Listwise deletion was used for the moderation analysis. Statistical significance was defined as *p* < .05.

## Results

On average, participants reported exposure to 1.26 ACEs (*SD* = 1.62). Fifty-seven percent of participants endorsed exposure to one or more ACEs (*n* = 38); 7.5% (*n* = 5) indicated exposure to four or more ACEs (Table 1). The mean burnout score was 2.70 (*SD* = .92). One in every five participants (20.9%; *n* = 14) reported significant levels of burnout, and 40.3% (*n* = 27) reported levels consistent with burnout ‘danger signs’. Roughly 38.8% (*n* = 26) of participants reported very low burnout. The mean resilience score was 6.49 (*SD* = 1.59). Number of ACEs, *F*(2,62) = .02, *p* = .98, burnout, *F*(2,64) = 2.11, *p* = .98, and resilience, *F*(2,56) = 2.68, *p* = .078, did not differ by occupation (see Table 3). Years working in the healthcare profession differed by occupation, *F*(2,59)=7.10, *p* = .002; specifically, medical assistants, *M* = 12.40, *SD* = 11.37, *p* = .004, and nurses, *M* = 14.25, *SD* = 4.92, *p* = .013, reported working more years in healthcare than physicians/residents, *M* = 5.27, *SD* = 5.55.

**Figure 1.**
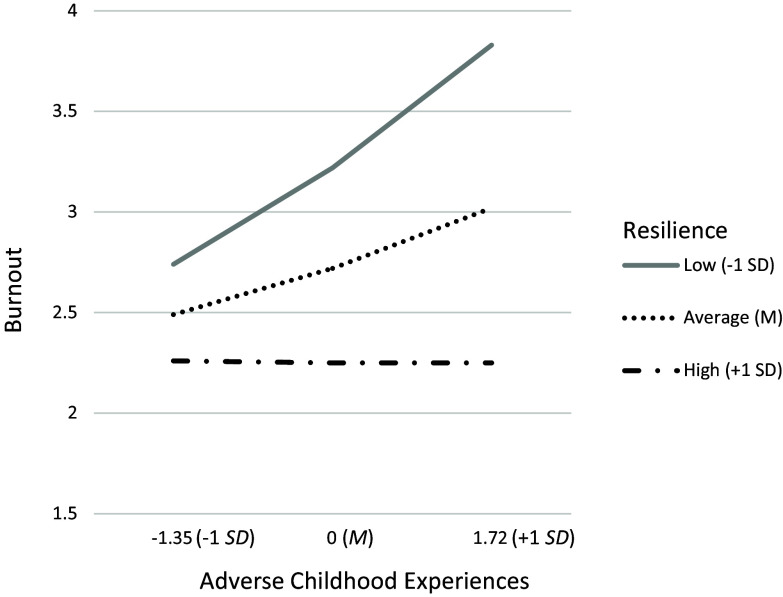
Interaction of adverse childhood experiences and resilience on health care provider burnout controlling for years in healthcare profession and occupation. *Note:* Low resilience, *β* = .36, *SE* = .11, *t* = 3.26, *p* = .002. Average resilience, *β* = .17, *SE* = .07, *t* = 2.58, *p* = .013. High resilience, *β* = −.003, *SE* = .10, *t* = −.03, *p* = .975.

At the bivariate level, ACE scores were positively associated with burnout (H1; *r* = .25, *p* = .048), and resilience was negatively associated with burnout (*r* = −.40, *p* = .002; Table 2). The moderation analysis revealed main effects of ACEs, *B* = .17, *SE* = .07, *p* = .013, and resilience, *B* = −.34, *SE*=.08, *p* = .000, on burnout, when controlling for number of years in the healthcare profession, *B* = .01, *SE* = .02, *p* = .72 (Table 4). There was a significant interaction of ACEs and resilience on burnout, *n* = 55, *B* = −.11, *SE* = .05, *p* = .029 (Figure 1). A positive relation was found between ACEs and burnout for participants who reported low, *t* = 3.21, *p* = .002, and average, *t* = 2.57, *p =* .013, levels of resilience. The association between ACEs and burnout was nonsignificant at high levels of resilience, *t* = .01, *p* = .99. A post-hoc moderation analysis including occupation as a covariate was also conducted; given lack of mean differences across variables, nurses and medical assistant were collapsed into one group [nurses/medical assistants = 1] and compared to physicians/residents [physicians/residents = 0]). Moderation findings did not meaningfully differ when occupation was included as a covariate in the model. See Table 4.

**Table 2.** Descriptive statistics and bivariate correlations

			*r*		
Variable	*M* (*SD*)	Range	Burnout	Resilience	Years
ACEs	1.26 (1.62)	.00–7.00	.25*	.07	.01
Burnout	2.70 (.92)	1.00–5.40	–	−.40**	.07
Resilience	6.49 (1.59)	.00–8.00	–	–	.17
Years of healthcare experience	7.00 (7.39)	1.00–32.00	–	–	–

*Note:* ACEs = Adverse Childhood Experiences; *n* = 59–67; *p < .05, **p <.01, ***p<.001.

**Table 3.** Group means and standard deviations by occupation

	Physicians/ residents (*n* = 44–50)		Medical assistants (*n* = 10–12)		Nurses (*n* = 4–5)		
Variable	*M*	*SD*	*M*	*SD*	*M*	*SD*	*F*(*df*)
Adverse childhood experiences	1.25	1.47	1.25	1.91	1.40	2.61	.02 (2,62) *p* = .981
Burnout	2.81	.85	2.53	1.16	2.00	.72	2.11 (2,64) *p* = .129
Resilience	6.25^a^	1.62	7.50^b^	1.08	6.60^b^	1.67	2.68 (2,56) *p* = .078
Years of experience in healthcare	5.27^a^	5.55	12.40^b^	11.37	14.25	4.92^b^	7.10** (2,59)

*Note*: means of groups with different subscripts are statistically different from one another; *p < .05, **p < .01, ***p <.001.

**Table 4.** Summary of moderation analysis for ACEs predicting burnout by resilience level

Model	*B*	*SE B*	*t*	LLCI-ULCI	*F*	*R* ^2^
Burnout					6.15***	.33
Constant	2.68	.17	16.25***	2.35–3.01		
Adverse childhood experiences	.17	.07	2.57*	.04–.31		
Resilience	−.34	.08	−4.33***	−.49–.18		
ACEs x resilience	−.11	.05	−2.25*	−.22–.01		
Years in healthcare profession	.01	.02	.31	−.03–.04		
Burnout (Post-Hoc)					5.31***	.35
Constant	2.71	.17	16.36***	2.38–3.05		
Adverse childhood experiences	.17	.07	2.58*	.04–.31		
Resilience	−.31	.08	−3.89***	−.47–−.15		
ACEs × resilience	−.12	.05	−2.31*	−.22- -.02		
Years in healthcare profession	.02	.02	.81	−.02–.05		
Occupation (physician/resident vs. nurse/medical assistant)	−.39	.31	−1.29	−1.01–.22		

*n* = 55; *p < .05, **p < .01, ***p < .001.

## Discussion

The current study assessed the relations among ACEs, resilience, and burnout among HCPs in primary care clinics at a safety-net hospital. While previous research has demonstrated that HCP burnout is positively associated with ACEs (Robertson *et al*., 2016; West *et al*., 2020; Clemens *et al*., 2021; Yellowlees *et al*., 2021; Williamson *et al*., 2025), but negatively associated with resilience (Cooke *et al*., 2013; McCain *et al*., 2018; Ferreira *et al*., 2021), this study is the first to examine resilience as a potential protective factor in the association between ACEs and burnout among HCPs in primary care.

In support of H1 and consistent with previously mentioned studies, HCPs who endorsed more ACEs reported higher levels of burnout. In addition, this association was moderated by resilience, even when controlling for the number of years in the healthcare profession (H2). More specifically, experiencing more ACEs correlated with experiencing greater burnout among HCPs with low-to-average resilience. For HCPs with higher levels of resilience, however, the association between ACEs and burnout was no longer significant. Overall, current findings suggest that the presence of resilience traits may help mitigate, or even prevent, burnout among all HCPs; however, it seems to be particularly beneficial for those with ACE histories.

Thus, it is imperative that hospital and clinic administrators emphasize the importance of building resilience among HCPs that provide care within the larger healthcare system (Shanafelt, 2021). Given few disclose psychological distress to their workplace (Zamir *et al*., 2022), healthcare organizations may benefit from following universal precaution as a trauma-informed approach (Substance Abuse and Mental Health Services Administration, 2014) by understanding the impact of ACEs on HCP well-being, identifying the signs and symptoms of trauma and burnout, and offering hospital-wide opportunities to enhance resilience. Perhaps, a viable path to decreased burnout and enhanced resilience involves routine, system-wide exposure to trauma-informed professional development or self-care opportunities (Sood *et al*., 2011). A specific intervention that has demonstrated particular effectiveness among HCPs is Mindfulness in Motion, an 8-week modified mindfulness-based intervention designed for nurses in chronically high-stress environments and to be delivered on-site, during work hours and in a group setting (Klatt *et al*., 2015). Notably, the effects of the intervention were sustained for 12 months (Klatt *et al*., 2022), suggesting adequate dosage could be achieved through annual participation. Even further, intentionally incorporating information on the link that exists between psychosocial experiences and overall health – in both patients and HCPs – may increase the trauma-informed nature of existing interventions, which is key for HCPs regularly interfacing with trauma-exposed populations. Notably, HCPs with ACE histories have more favourable attitudes towards trauma-informed care (Selwyn *et al*., 2023), highlighting the importance of attracting and retaining HCPs with lived experiences, especially in organizations dedicated to building a resilient workforce.

When considering findings, certain limitations must be taken into account. For example, this study used a cross-sectional design, which prohibits causal conclusions regarding the directionality of the association between ACEs and burnout. Also, this study only measured ACEs among HCPs; however, many other types of traumatic events can influence provider burnout, such as workplace violence (Mento *et al*., 2020) and secondary trauma (Leung *et al*., 2023). Other limitations concern the small sample sizes, especially in relation to medical assistant and nurse participants, as well as the need to collapse physicians/residents into one group due to a subset of current residents selecting ‘physician’ as their occupation. Replication in larger samples with more sizable and distinct groups of residents, physicians, nurses, and medical assistants is needed.

## Conclusions

While the precise definition of resilience is still debated and biomedical research on resilience is in its nascent phase, these findings indicate that resilience is particularly important for HCP well-being, increasing capacities to bounce back amidst the rigours of highly intense, demanding, and emotionally challenging work. Such implications are especially important for HCPs working in safety-net or resource-limited settings as well as those with higher cumulative ACEs. As research continues to elucidate risk factors associated with burnout – such as stressful work environments and ACEs – promoting resilience will be of increasing importance both individually and institutionally (Figueroa and Jha, 2018). Consequently, further research is needed to understand how resilience can be cultivated and supported. Finally, it is important to note that an imbalanced overemphasis of resilience without interrogating and understanding the external conditions that demand its presence can be highly problematic and inadvertently deflect attention from necessary system-level reform. In healthcare, therefore, promoting resilience should not replace institutional efforts to mitigate burnout nor be used to justify or maintain unsafe cultures and policies.

## References

[ref1] Agency for Healthcare Research and Quality (2023) National Healthcare Quality and Disparities Report. Available at https://www.ahrq.gov/research/findings/nhqrdr/nhqdr23/index.html (accessed 20 March 2025).

[ref2] Alrawashdeh HM , Al-Tammemi AB , Alzawahreh MK , Al-Tamimi A , Elkholy M , Al Sarireh F , Abusamak M , Elehamer NMK , Malkawi A , Al-Dolat W , Abu-Ismail L , Al-Far A and Ghoul I (2021) Occupational burnout and job satisfaction among physicians in times of COVID-19 crisis: a convergent parallel mixed-method study. BMC Public Health 21(1), 811. 10.1186/s12889-021-10897-4. 33906619 PMC8079229

[ref3] Anagnostopoulos F , Liolios E , Persefonis G , Slater J , Kafetsios K and Niakas D (2012) Physician burnout and patient satisfaction with consultation in primary health care settings: evidence of relationships from a one-with-many design. Journal of Clinical Psychology in Medical Settings 19(4), 401–410. 10.1007/s10880-011-9278-8. 22327237

[ref4] Bridgeman PJ , Bridgeman MB and Barone J (2018) Burnout syndrome among healthcare professionals. American Journal of Health-System Pharmacy 75(3), 147–152. 10.2146/ajhp170460. 29183877

[ref5] Clemens V , Beschoner P , Jarczok MN , Weimer K , Kempf M , Morawa E , Geiser F , Albus C , Steudte-Schmiedgen S , Gündel H and Fegert JM (2021) The mediating role of COVID-19-related burden in the association between adverse childhood experiences and emotional exhaustion: results of the egePan–VOICE study. European Journal of Psychotraumatology 12(1), 1976441. 34621498 10.1080/20008198.2021.1976441 PMC8491662

[ref6] Connor KM and Davidson JRT (2003) Development of a new resilience scale: the Connor-Davidson Resilience Scale (CD-RISC). Depression and Anxiety 18(2), 76–82. 10.1002/da.10113. 12964174

[ref7] Cooke GPE , Doust JA and Steele MC (2013) A survey of resilience, burnout, and tolerance of uncertainty in Australian general practice registrars. BMC Medical Education 13(2), 1–6. 10.1186/1472-6920-13-2. 23294479 PMC3563610

[ref8] Dewa CS , Loong D , Bonato S , Thanh NX and Jacobs P (2014) How does burnout affect physician productivity? A systematic literature review. BMC Health Services Research 14, 325. 10.1186/1472-6963-14-325. 25066375 PMC4119057

[ref9] Dyrbye LN , Shanafelt TD , Sinsky CA , Cipriano PF , Bhatt J , Ommaya A , West CP and Meyers D (2017) Burnout among healthcare professionals: a call to explore and address this underrecognized threat to safe, high-quality care. NAM Perspectives 7(7), 1–11. 10.31478/201707b.

[ref10] Felitti VJ , Anda RF , Nordenberg D , Williamson DF , Spitz AM , Edwards V , Koss MP and Marks JS (1998) Relationship of childhood abuse and household dysfunction to many of the leading causes of death in adults: the Adverse Childhood Experiences (ACE) study. American Journal of Preventive Medicine 14(4), 245–258. 10.1016/s0749-3797(98)00017-8. 9635069

[ref11] Ferreira M , Marques A and Gomes PV (2021) Individual resilience interventions: a systematic review in adult population samples over the last decade. International Journal of Environmental Research and Public Health 18(14), 7564. 10.3390/ijerph18147564. 34300018 PMC8307772

[ref12] Figueroa JF and Jha AK (2018) Approach for achieving effective care for high-need patients. JAMA Internal Medicine 178(6), 845–846. 29630697 10.1001/jamainternmed.2018.0823

[ref13] Han S , Shanafelt TD , Sinsky CA , Awad KM , Dyrbye LN , Fiscus LC , Trockel M and Goh J (2019) Estimating the attributable cost of physician burnout in the United States. Annals of Internal Medicine 170(11), 784–790. 10.7326/M18-1422. 31132791

[ref16] Khullar D (2023) Burnout, professionalism, and the quality of US health care. JAMA Health Forum 4(3), e230024. 10.1001/jamahealthforum.2023.0024. 36961455

[ref14] Klatt M , Steinberg B and Duchemin AM (2015) Mindfulness in Motion (MIM): an onsite mindfulness-based intervention (MBI) for chronically high-stress work environments to increase resiliency and work engagement. Journal of Visualized Experiments 101, e52359. 10.3791/52359. PMC4545050 26168365

[ref15] Klatt M , Westrick A , Bawa R , Gabram O , Blake A and Emerson B (2022) Sustained resiliency building and burnout reduction for healthcare professionals via organizational sponsored mindfulness programming. Explore 18(2), 179–186. 33931362 10.1016/j.explore.2021.04.004

[ref17] Lathan EC , Haynes T , Langhinrichsen-Rohling R , Sonu SC and Powers A (2023) Primary care providers’ knowledge, perceptions, and practice of trauma-informed care in a public healthcare setting. Family and Community Health 46(4), 209–219. 10.1097/FCH.0000000000000376. 37703510

[ref18] Leung T , Schmidt F and Mushquash C (2023) A personal history of trauma and experience of secondary traumatic stress, vicarious trauma, and burnout in mental health workers: a systematic literature review. Psychological Trauma: Theory, Research, Practice, and Policy 15(S2), S213–S222. 35511539 10.1037/tra0001277

[ref19] Li CJ , Shah YB , Harness ED , Goldberg ZN and Nash DB (2023) Physician burnout and medical errors: exploring the relationship, cost, and solutions. American Journal of Medical Quality 38(4), 196–202. 37382306 10.1097/JMQ.0000000000000131

[ref51] Malach-Pines A (2005). The burnout measure, short version. International Journal of Stress Management 12(1), 78–88. 10.1037/1072-5245.12.1.78.

[ref20] Mangory KY , Ali LY , Rø KI and Tyssen R (2021) Effect of burnout among physicians on observed adverse patient outcomes: a literature review. BMC Health Services Research 21(1), 369. 10.1186/s12913-021-06371-x. 33879135 PMC8057942

[ref21] Maslach C , Jackson SE and Leiter MP (1997) Maslach burnout inventory. In Evaluating Stress: A Book of Resources, 3rd Edn. Lanham: Scarecrow Education, pp. 191–218.

[ref22] Maunder RG , Lancee WJ , Mae R , Vincent L , Peladeau N , Beduz MA , Hunter JJ and Leszcz M (2010) Computer-assisted resilience training to prepare healthcare workers for pandemic influenza: a randomized trial of the optimal dose of training. BMC Health Services Research 10, 72. 10.1186/1472-6963-10-72. 20307302 PMC2851711

[ref23] McCain RS , McKinley N , Dempster M , Campbell WJ and Kirk SJ (2018) A study of the relationship between resilience, burnout and coping strategies in doctors. Postgraduate Medical Journal 94(1107), 43–47. 10.1136/postgradmedj-2016-134683 28794171

[ref24] Mento C , Silvestri MC , Bruno A , Muscatello MRA , Cedro C , Pandolfo G and Zoccali RA (2020) Workplace violence against healthcare professionals: a systematic review. Aggression and Violent Behavior, 51, 101381.

[ref25] Mercer L , Cookson A , Simpson-Adkins G and van Vuuren J (2023) Prevalence of adverse childhood experiences and associations with personal and professional factors in health and social care workers: a systematic review. Psychological Trauma: Theory, Research, Practice, and Policy 15(Suppl 2), S231–S245. 10.1037/tra0001506. 37141025

[ref26] Meredith LS , Bouskill K , Chang J , Larkin J , Motala A and Hempel S (2022) Predictors of burnout among US healthcare providers: a systematic review. BMJ Open 12(8), e054243. 10.1136/bmjopen-2021-054243. PMC9422884 36008065

[ref27] Nigam JA , Barker RM , Cunningham TR , Swanson NG and Chosewood LC (2023) Vital signs: health worker-perceived working conditions and symptoms of poor mental health — quality of worklife survey, United States, 2018–2022. MMWR Morbidity and Mortality Weekly Report 72, 1197–1205. 10.15585/mmwr.mm7244e1. 37917563 PMC10629752

[ref28] Owoc J , Mańczak M , Jabłońska M , Tombarkiewicz M and Olszewski R (2022) Association between physician burnout and self-reported errors: meta-analysis. Journal of Patient Safety 18(1), e180–e188. 10.1097/PTS.0000000000000724. 34951608

[ref29] Pines A and Aronson E (1988) Career Burnout: Causes and Cures. New York: Free Press.

[ref30] Popescu I , Fingar KR , Cutler E , Guo J and Jiang HJ (2019) Comparison of 3 safety-net hospital definitions and association with hospital characteristics. JAMA Network Open 2(8), e198577. 10.1001/jamanetworkopen.2019.8577. 31390034 PMC6686776

[ref31] Powers A , Langhinrichsen-Rohling R , Sonu SC , Haynes T and Lathan EC (2024). Brief trauma-informed care training to enhance primary care providers’ knowledge, comfort, and implementation of trauma-informed care: a pilot effectiveness study. Psychological Services 21(4), 792–796. 10.1037/ser0000823. 37956055 PMC11089069

[ref33] Renkiewicz GK and Hubble MW (2021) Secondary traumatic stress in emergency services systems (STRESS) project: quantifying personal trauma profiles for secondary stress syndromes in emergency medical services personnel with prior military service. Journal of Special Operations Medicine 21(1), 55–64. 10.55460/AO3Y-HY3W. 33721308

[ref32] Renkiewicz GK and Hubble MW (2023) Secondary trauma response in emergency services systems (STRESS) project: quantifying and predicting vicarious trauma in emergency medical services personnel. British Paramedic Journal 7(4), 23–34. 10.29045/14784726.2023.3.7.4.23. 36875827 PMC9983063

[ref34] Robertson HD , Elliott AM , Burton C , Iversen L , Murchie P , Porteous T and Matheson C (2016) Resilience of primary healthcare professionals: a systematic review. British Journal of General Practice 66(647), e423–e433. 10.3399/bjgp16X685261 PMC4871308 27162208

[ref35] Selwyn CN , Lathan EC , Platt T and Minchew L (2023) How healthcare providers reconcile bad things happening to good patients: the role of just world beliefs in attitudes toward trauma-informed care. Journal of Trauma and Dissociation 24(5), 640–654. 36987779 10.1080/15299732.2023.2195404

[ref37] Shanafelt TD , Mungo M , Schmitgen J , Storz KA , Reeves D , Hayes SN , Sloan JA , Swensen SJ and Buskirk SJ (2016) Longitudinal study evaluating the association between physician burnout and changes in professional work effort. Mayo Clinic Proceedings 91(4), 422–431. 10.1016/j.mayocp.2016.02.001. 27046522

[ref36] Shanafelt TD (2021) Physician well-being 2.0: where are we and where are we going?. Mayo Clinic Proceedings 96, 2682–2693. 10.1016/j.mayocp.2021.06.005. 34607637

[ref38] Sood A , Prasad K , Schroeder D and Varkey P (2011) Stress management and resilience training among Department of Medicine faculty: a pilot randomized clinical trial. Journal of General Internal Medicine 26(8), 858–861. 10.1007/s11606-011-1640-x. 21279454 PMC3138987

[ref39] Southwick SM , Bonanno GA , Masten AS , Panter-Brick C and Yehuda R (2014) Resilience definitions, theory, and challenges: interdisciplinary perspectives. European Journal of Psychotraumatology 5, 25338. 10.3402/ejpt.v5.25338. PMC4185134 25317257

[ref40] Stork BR , Akselberg NJ , Qin Y and Miller DC (2020) Adverse childhood experiences (ACEs) and community physicians: what we’ve learned. TPJ 24(2), 19.099. 10.7812/TPP/19.099. PMC7021137 32069204

[ref41] Substance Abuse and Mental Health Services Administration (2014) SAMHSA’s Concept of Trauma and Guidance for a Trauma-Informed Approach. Rockville, MD: US Department of Health and Human Services.

[ref42] Tawfik DS , Profit J , Morgenthaler TI , Satele DV , Sinsky CA , Dyrbye LN , Tutty MA , West CP and Shanafelt TD (2018) Physician burnout, well-being, and work unit safety grades in relationship to reported medical errors. Mayo Clinic Proceedings 93(11), 1571–1580. 10.1016/j.mayocp.2018.05.014. 30001832 PMC6258067

[ref45] Vaishnavi S , Connor K and Davidson JRT (2007) An abbreviated version of the connor-Davidson Resilience Scale (adverse), the CD-RISC2: psychometric properties and applications in psychopharmacological trials. Psychiatry Research 152(2–3), 293–297. 10.1016/j.psychres.2007.01.006. 17459488 PMC2041449

[ref46] West CP , Dyrbye LN , Sinsky C , Trockel M , Tutty M , Nedelec L , Carlasare LE and Shanafelt TD (2020) Resilience and burnout among physicians and the general US working population. JAMA Network Open 3(7), e209385. 32614425 10.1001/jamanetworkopen.2020.9385 PMC7333021

[ref43] West CP , Dyrbye LN and Shanafelt TD (2018) Physician burnout: contributors, consequences and solutions. Journal of Internal Medicine 283(6), 516–529. 10.1111/joim.12752. 29505159

[ref44] Williams BW , Welindt D , Hafferty FW , Stumps A , Flanders P and Williams MV (2021) Adverse childhood experiences in trainees and physicians with professionalism lapses: implications for medical education and remediation. Academic Medicine 96(5), 736–743. 10.1097/ACM.0000000000003532. 32520753

[ref47] Williamson L , Daniel SS , Carter J , Ridenhour A , Pulgar CA , Gay Y and Debinski B (2025) Negative effects of adverse childhood experiences and absence of positive childhood experiences on healthcare employees: survey findings built on 10 years of trauma-informed development. Front Public Health 12, 1494587. 10.3389/fpubh.2024.1494587. 39835305 PMC11743665

[ref48] Wisetborisut A , Angkurawaranon C , Jiraporncharoen W , Uaphanthasath R and Wiwatanadate P (2014) Shift work and burnout among health care workers. Occupational Medicine (London) 64(4), 279–286. 10.1093/occmed/kqu009. 24550196

[ref49] Yellowlees P , Coate L , Misquitta R , Wetzel AE and Parish MB (2021) The association between adverse childhood experiences and burnout in a regional sample of physicians. Academic Psychiatry 45(2), 159–163. 10.1007/s40596-020-01381-z. 33409937

[ref50] Zamir A , Tickle A and Sabin-Farrell R (2022) A systematic review of the evidence relating to disclosure of psychological distress by mental health professionals within the workplace. Journal of Clinical Psychology 78(9), 1712–1738. 10.1002/jclp.23339. 35247268 PMC9541467

